# PhenoScanner V2: an expanded tool for searching human genotype–phenotype associations

**DOI:** 10.1093/bioinformatics/btz469

**Published:** 2019-06-24

**Authors:** Mihir A Kamat, James A Blackshaw, Robin Young, Praveen Surendran, Stephen Burgess, John Danesh, Adam S Butterworth, James R Staley

**Affiliations:** 1 MRC/BHF Cardiovascular Epidemiology Unit, Department of Public Health and Primary Care, University of Cambridge, Cambridge CB1 8RN, UK; 2 MRC Biostatistics Unit, University of Cambridge, Cambridge CB2 0SR, UK; 3 Wellcome Trust Sanger Institute, Hinxton CB10 1SA, UK; 4 NIHR Blood and Transplant Research Unit, Department of Public Health and Primary Care, University of Cambridge, Cambridge CB1 8RN, UK; 5 MRC Integrative Epidemiology Unit, Population Health Sciences, Bristol Medical School, University of Bristol, Bristol BS8 2BN, UK

## Abstract

**Summary:**

PhenoScanner is a curated database of publicly available results from large-scale genetic association studies in humans. This online tool facilitates ‘phenome scans’, where genetic variants are cross-referenced for association with many phenotypes of different types. Here we present a major update of PhenoScanner (‘PhenoScanner V2’), including over 150 million genetic variants and more than 65 billion associations (compared to 350 million associations in PhenoScanner V1) with diseases and traits, gene expression, metabolite and protein levels, and epigenetic markers. The query options have been extended to include searches by genes, genomic regions and phenotypes, as well as for genetic variants. All variants are positionally annotated using the Variant Effect Predictor and the phenotypes are mapped to Experimental Factor Ontology terms. Linkage disequilibrium statistics from the 1000 Genomes project can be used to search for phenotype associations with proxy variants.

**Availability and implementation:**

PhenoScanner V2 is available at www.phenoscanner.medschl.cam.ac.uk.

## 1 Introduction

Dense array-based human genetic studies, such as genome-wide association studies (GWAS), have identified many thousands of associations between genetic variants and a diverse set of phenotypes. The challenge now facing the human genomics community is to understand the mechanisms underlying these associations. One approach to aid biological insight into disease mechanisms is to cross-reference genetic associations across a range of phenotypes, including disease states, cellular traits and other intermediate traits. To enable such ‘phenome scans’ we developed the online tool PhenoScanner (Staley *et al.*, 2016). Since its release in 2016, PhenoScanner has been accessed by hundreds of users to assist a range of analyses from analyses linking proteins to disease (Sun *et al.*, 2018) to interrogating novel loci associated with blood cell phenotypes (Astle *et al.*, 2016).

In recent years, there has been a rapid expansion in the availability of genetic association statistics with the maturation of genetic biobanks with rich phenotypic information. Moreover, the scope of molecular phenotypes in genetic association studies has increased with the publication of multi-tissue gene expression GWAS (GTEx Consortium *et al.*, 2017) and GWAS of thousands of plasma proteins (Sun *et al.*, 2018). However, integrating genetic associations across this vast array of data sources remains challenging. Hence, to facilitate improved ‘phenome scans’, we have released an updated version of PhenoScanner (PhenoScanner V2) with new features including: (i) an expanded database of human genotype–phenotype associations associations split into phenotype classes (diseases and traits, gene expression, proteins, metabolites and epigenetics); (ii) additional search options including gene, genomic region and phenotype-based queries; (iii) linkage disequilibrium (LD) information for the five super-ancestries in 1000 Genomes; (vi) variant annotation and trait ontology mappings and (v) a brand new web interface and API.

## 2 Materials and methods

PhenoScanner V2 consists of a Python-R interface which connects to a series of MySQL databases. To develop the catalogue of human genotype–phenotype associations, we identified and collated >5000 genetic association datasets from publicly available lists of full summary associations results compiled by the NHGRI-EBI (https://www.ebi.ac.uk/gwas/downloads/summary-statistics) and NHLBI (https://grasp.nhlbi.nih.gov/FullResults.aspx), as well as from recent literature reviews and lists of omics GWAS (e.g. Sun *et al.*, 2018 for protein levels). The catalogue currently contains results for diseases and traits (∼30 billion associations), gene expression (∼84 million associations), protein levels (∼35 billion associations), metabolite levels (∼3 billion associations) and epigenetic markers (∼13 million associations). To ensure consistent formatting across datasets, all of the variants were aligned to the NCBI plus strand, rsIDs were updated to dbSNP 147 (Sherry *et al.*, 2001) and chromosome-positions [GRCh37 (hg19) and GRCh38 (hg38)] were added or updated using dbSNP 147 and liftOver (https://genome.ucsc.edu/cgi-bin/hgLiftOver). LD measures between neighbouring variants in the autosomal chromosomes were calculated using phased haplotypes for the five super-ancestries (European, African, Admixed American, East Asian and South Asian) in the 1000 Genomes Project phase 3 (1000 Genomes Project Consortium *et al.*, 2015). We calculated *D*' and *r*^2^ for pairs of variants within 500 Kb and kept LD statistics with *r*^2^ ≥ 0.5. All phenotypes were mapped to Experimental Factor Ontology terms (Malone *et al.*, 2010) using ZOOMA (https://www.ebi.ac.uk/spot/zooma/). Variant and gene annotation for all of the variants was performed using Ensembl Variant Effect Predictor V88 (McLaren *et al.*, 2016) with GENCODE transcripts V26 (Harrow *et al.*, 2012) mapped to build 37 positions. Nearest genes for intergenic variants were retrieved using the BEDOPS tool version 2.4.26 (Neph *et al.*, 2012).

Users may enter one genetic variant, gene, genomic region or trait into the text box on the home page (www.phenoscanner.medschl.cam.ac.uk) or upload up to 100 genetic variants, 10 genes or 10 genomic regions as a tab-delimited text file. PhenoScanner V2 also has an API with an associated R package and Python command line tool (www.phenoscanner.medschl.cam.ac.uk/tools), allowing users to search for genotype–phenotype associations from PhenoScanner V2 inside R or from a terminal. When querying genetic variants, all results regardless of *P*-value can be displayed allowing the user to identify evidence against associations with phenotypes. To produce manageable results sets, only results with *P *<* *1 × 10^−5^ are returned for queries of genes, genomic regions or phenotypes. Once a query is evoked, the Python-R interface annotates the genetic variant, gene, genomic region or phenotype using dbSNP (or ZOOMA for trait queries), before searching the requested association databases and filtering the results based on the specified *P*-value threshold. The new web interface then presents the results and makes them available to download. All associations for each genetic variant are aligned such that the effect allele is the same across all results. The associations with proxy variants are aligned such that their effect alleles are given with respect to the effect allele of the corresponding queried variant.

## 3 Results

To demonstrate the value of the expanded database and additional functionality of PhenoScanner V2, we searched for ‘rs10840293’, ‘SWAP70’ and ‘coronary heart disease’. PhenoScanner V2 found >150 000 results with rs10840293 (variant annotation: intronic variant in *SWAP70*) or one of its proxies (*r*^2^ ≥ 0.8 in Europeans), more than 100 times the number of associations found for the same variant query using PhenoScanner V1 (1405 associations); the NHGRI-EBI GWAS Catalog (MacArthur *et al.*, 2017) only returns four results for rs10840293. In particular, PhenoScanner V2 identifies strong associations of rs10840293 with coronary heart disease (van der Harst and Verweij, 2018), blood pressure (https://www.nealelab.is/uk-biobank) and platelet width (Astle *et al.*, 2016), as well as with whole blood gene expression (Võsa *et al*., 2018) and plasma protein levels (Sun *et al.*, 2018) of SWAP70 (all with *P *<* *5 × 10^−8^), suggesting a possible blood pressure related mechanism affecting coronary heart disease risk at this locus potentially regulated via *SWAP70* expression. Variants in the *SWAP70* gene had >6000 associations with *P *<* *1 × 10^−5^ (compared with 27 associations found by the GWAS Catalog), while there were >50 000 genetic associations with coronary heart disease with *P *<* *1 × 10^−5^ across the genome (compared with 1092 associations found by the GWAS Catalog).

## 4 Conclusion

PhenoScanner V2 is a large curated database of human genotype–phenotype associations from publicly available genetic association studies. This catalogue of results greatly extends PhenoScanner V1 in both scale and phenotypic breadth, with tables of genetic associations for diseases and traits, gene expression, protein levels, metabolites levels and epigenetic markers. PhenoScanner V2 also has additional annotation and functionality. The database can now be searched for genes, genomic regions and traits, while variant annotation, phenotype ontology mappings and LD statistics from a wider range of ethnic groups have been incorporated to enhance utility and interpretation.

## Funding

This work was supported by the UK Medical Research Council [G0800270, MR/L003120/1]; the British Heart Foundation [SP/09/002, RG/13/13/30194, RG/18/13/33946]; Pfizer [G73632]; the European Research Council [268834]; the European Commission Framework Programme 7 [HEALTH-F2-2012-279233]; the National Institute for Health Research; and Health Data Research UK. The views expressed are those of the authors and not necessarily those of the NHS or the NIHR.

*Conflict of Interest*: none declared.
